# Differential Modulation of GABA_A_ and NMDA Receptors by an α7-nicotinic Acetylcholine Receptor Agonist in Chronic Glaucoma

**DOI:** 10.3389/fnmol.2017.00422

**Published:** 2017-12-18

**Authors:** Xujiao Zhou, Yuan Zong, Rong Zhang, Xuejin Zhang, Shenghai Zhang, Jihong Wu, Xinghuai Sun

**Affiliations:** ^1^Eye Institute, Eye and ENT Hospital, State Key Laboratory of Medical Neurobiology, Institutes of Brain Science and Collaborative Innovation Center for Brain Science, Shanghai Medical College, Fudan University, Shanghai, China; ^2^Shanghai Key Laboratory of Visual Impairment and Restoration, Fudan University, Shanghai, China; ^3^Key Laboratory of Myopia, Ministry of Health, Shanghai, China; ^4^Department of Ophthalmology and Vision Science, Eye and ENT Hospital, Fudan University, Shanghai, China

**Keywords:** α7-nAChR, GABA_A_ receptors, NMDA receptors, neuroprotection, chronic glaucoma

## Abstract

Presynaptic modulation of γ-aminobutyric acid (GABA) release by an alpha7 nicotinic acetylcholine receptor (α7-nAChR) agonist promotes retinal ganglion cell (RGC) survival and function, as suggested by a previous study on a chronic glaucomatous model from our laboratory. However, the role of excitatory and inhibitory amino acid receptors and their interaction with α7-nAChR in physiological and glaucomatous events remains unknown. In this study, we investigated GABA_A_ and N-methyl-D-aspartate (NMDA) receptor activity in control and glaucomatous retinal slices and the regulation of amino acid receptor expression and function by α7-nAChR. Whole-cell patch-clamp recordings from RGCs revealed that the α7-nAChR specific agonist PNU-282987 enhanced the amplitude of currents elicited by GABA and reduced the amplitude of currents elicited by NMDA. The positive modulation of GABA_A_ receptor and the negative modulation of NMDA receptor (NMDAR) by PNU-282987-evoked were prevented by pre-administration of the α7-nAChR antagonist methyllycaconitine (MLA). The frequency and the amplitude of glutamate receptor-mediated miniature glutamatergic excitatory postsynaptic currents (mEPSCs) were not significantly different between the control and glaucomatous RGCs. Additionally, PNU-282987-treated slices showed no alteration in the frequency or amplitude of mEPSCs relative to control RGCs. Moreover, we showed that expression of the α1 subunit of the GABA_A_ receptor was downregulated and the expression of the NMDAR NR2B subunit was upregulated by intraocular pressure (IOP) elevation, and the changes of high IOP were blocked by PNU-282987. In conclusion, retina GABA_A_ and NMDARs are modulated positively and negatively, respectively, by activation of α7-nAChR in *in vivo* chronic glaucomatous models.

## Introduction

Interactions between the cholinergic, γ-aminobutyric acidergic (GABAergic) and glutamatergic systems, as the major neurotransmitters in the central nervous system (CNS), are involved in many neurodevelopmental, neurological and neurodegenerative disorders (Mittag et al., 2000; Sattler and Tymianski, 2001; Lin et al., 2014a; Lewerenz and Maher, 2015). Acetylcholine (ACh) reduces the N-methyl-d-aspartate (NMDA) receptor (NMDAR)-mediated currents by acting on muscarinic and nicotinic receptors in primary neocortical neuronal cultures (Aizenman et al., 1991), the rat neocortex (Flores-Hernandez et al., 2009) and the retina (O’Dell and Christensen, 1988). Alpha7 nicotinic acetylcholine receptor (α7-nAChR)–knockout mice exhibit reduced cortical NMDARs and glutamatergic synapses (Lin et al., 2014b) and decreased cortical levels of GABAergic markers (Liu et al., 2006; Lin et al., 2014a). The interaction among the presynaptic α7-nAChR (Dougherty et al., 2003), glutamatergic (Schaeffer and Gattaz, 2008) and GABAergic (Spencer et al., 2006) systems may be involved in the early stages of Alzheimer’s disease.

In the mammalian retina, starburst amacrine cells are defined at the molecular level as the only cholinergic neurons (Seung and Sümbül, 2014). Homopentameric α7-nAChR is abundantly expressed in retinal amacrine, bipolar, and ganglion cells in rabbits and mice (Keyser et al., 2000; Strang et al., 2005; Dmitrieva et al., 2007; Zhou et al., 2017). Alpha7-nAChR agonists PNU-282987 can prevent retinal ganglion cells (RGCs) death caused by glutamate excitotoxicity in cultured retinal RGCs and have the neuron protective effects against loss of RGCs in an *in vivo* glaucoma model (Zhang and Berg, 2007; Zhou et al., 2017). Notably, the major neurotransmitters, GABA and glutamate, are also relevant to ocular diseases through excessive neuronal excitability resulting from the hypofunction of inhibitory signaling or the overexpression of NMDARs (Shareef et al., 1995; Mittag et al., 2000; Sattler and Tymianski, 2001; Dmitrieva et al., 2007; Chen et al., 2011; Seung and Sümbül, 2014; Li et al., 2017). In glaucoma, the chronic exposure of neurons to NMDA promotes the neurodegenerative disease process (Ullian et al., 2004; Lewerenz and Maher, 2015), and allopregnanolone potentiates the activity of GABA_A_ receptors, thereby having a neuroprotective effect (Ishikawa et al., 2014). Our prior studies have shown deficits in α7-nAChR expression accompanied by downregulation of GABA-synthesizing enzyme glutamic acid decarboxylase 65/67 and GABA in the retinas of a chronic rat glaucoma model (Zhou et al., 2017). However, the role of postsynaptic amino acid receptors and their interaction with α7-nAChR in physiological and glaucomatous events remains unknown. To examine the neuronal circuit changes associated with glaucoma in greater detail, the relationship between α7-nAChR and amino acid receptors in the retina must be explored.

To investigate the glaucoma-associated neuronal changes, we used patch-clamp recordings, Western blotting and immunostaining to first explore whether the RGC postsynaptic receptors are affected by glaucoma, with a focus on the GABA_A_α1 receptor and NMDARs. In addition, we further investigated the modulatory effects of PNU-282987 to understand the cholinergic modulatory effects in glaucoma.

## Materials and Methods

### Ethics

All experimental procedures conformed to the ARVO Statement for the Use of Animals in Ophthalmic and Vision Research and adhered to the guidelines for the Care and Use of Laboratory Animals formulated by the Animal Ethical Committee of Fudan University (Zhou et al., 2017). Animals were maintained in a 12 h light/dark cycle environment, temperatures at 23 ± 2°C, and humidity at 60%–70%. All efforts were made to minimize animal suffering and to reduce the number of animals used. All procedures and experimental protocols conducted on the animals were approved by the Institutional Animal Care and Ethics Committee of Fudan University (see the ethics certificate).

### Animals

Detailed steps as described in our previous articles (Zhou et al., 2017). Adult male Wistar rats (weighing 200–250 g; 2 months of age; SLAC Laboratory Animal Co., Ltd., Shanghai, China) were used.

### Rat Model of Ocular Hypertension

Detailed steps as described in our previous articles (Wu et al., 2010; Chen et al., 2011; Zhou et al., 2017), episcleral veins of the right eye were cauterized. The contralateral eye gone through a sham operation. Intraocular pressure (IOP) value was measured using a tonometer. Experiments were performed within 3 weeks following episcleral vein cauterization (EVC); age-matched normal animals served as controls.

### Preparation of Retinal Slices

The eyeball of rats were transferred to oxygenated sucrose, ice-cold cutting solution contained (in mM): 124 sucrose, 3 KCl, 1.25 NaH_2_PO_4_, 26 NaHCO_3_, 10 glucose, 0.2 CaCl_2_, 3.8 MgCl_2_, 3 sodium pyruvate (pH 7.4). Retinal slices were cut to 200 μm thick using a manual slicer and were allowed to rest for 40 min before recording (Zhou et al., 2017).

### Electrophysiology Recording

As previously described (Li et al., 2017; Zhou et al., 2017), GABA or NMDA (100 μM NMDA + 5 μM glycine) was applied for 8 s using the pressure feeding system (Picospritzer, General Valve Corp., Fairfield, NJ, USA) through a small-tipped (2 μm) pipette that was moved under visual control to within 50–100 μm from the recording neuron soma.

To record the GABA-induced whole-cell current, the pipette solution contained (in mM): 150 CsCl, 0.4 GTP-Na, 4 ATP-Mg, 10 HEPES, 1 EGTA, 0.1 CaCl_2_, 1 MgCl_2_, pH 7.2 adjusted with CsOH. GABA-induced current was induced in the presence of CNQX (10 μM), D-AP5 (10 μM), strychnine (1 μM) and the Na^+^-channel blocker tetrodotoxin (TTX, 1 μM), in the control solution.

To record the NMDA-induced whole-cell current, the patch pipettes contained (in mM): 125 CsCH_3_SO_3_, 1 MgCl_2_, 10 HEPES, 15 tetraethylammonium chloride (TEA-Cl), 0.5 GTP-Na, 4 ATP-Mg, 12 phosphocreatine, pH 7.25 adjusted with CsOH. ACSF (in mM): 125 NaCl, 3 KCl, 2 CaCl_2_, 1 MgCl_2_, 15 glucose, 1.25 NaH_2_PO_4_, 26 NaHCO_3_, 0.005 s trychnine and 0.02 SR95531 (pH 7.4). After patch-clamp recording was established, ACSF component changes to 125 NaCl, 3 KCl, 2 CaCl_2_, 15 glucose, 1.25 NaH_2_PO_4_, 26 NaHCO_3_, 0.02 ascorbic acid. NMDA-gated current (I_NMDA_) was induced after preincubation with CNQX (10 μM), TTX (1 μM), SR95531 (10 μM) and strychnine (1 μM).

Excitatory postsynaptic currents (EPSCs) were recorded in the whole-cell configuration in voltage-clamp mode (mM): 120 CsMeSO_3_, 0.2 GTP-Na, 2 ATP-Mg, 5 NaCl, 2 EGTA, 10 HEPES, 10 TEA-Cl, pH 7.2 adjusted with CsOH, 275 mOsm/l. In order to block the rapid Na+ currents, lidocaine N-ethyl bromide (QX314, 2.0 mM) was added to the pipette solution.

### Data Analysis

Detailed steps as described in our previous articles (Zhou et al., 2017). Using an Axopatch-Multiclamp 700B Amplifier and a Digidata 1440A system.

### Drug Administration

As previously described (Zhou et al., 2017), rats received an intravitreal injection of 5 μl PNU-282987 (100 μM) once a week. The contralateral eye received intravitreal injections of 5 μL phosphate-buffered saline. CNQX (10 μM) and D-AP5 (50 μM) to inhibit ionotropic glutamate receptors, TTX (1 μM) to abolish spontaneous action potentials, ifenprodil (10 μM) to inhibit NR2B-containing NMDAR, strychnine (1 μM) to block glycine receptors and SR95531 (10 μM) to block GABA_A_ receptors. methyllycaconitine (MLA; 100 nM) was applied 15 min prior to and during drugs application to block α7-nAChR. All drugs were purchased from Sigma-Aldrich.

### Western Blotting

Detailed steps as described in our previous articles (Wu et al., 2010; Zhou et al., 2017). The primary antibodies: rabbit polyclonal antibody against GABA_A_ alpha 1 (ab33299, 1:1000; Abcam, Cambridge, MA, USA), rabbit monoclonal antibody against NMDAR2B (4212S, 1:1000; Cell Signaling Technology, Danvers, MA, USA), rabbit monoclonal antibody against NMDAR1 (#5704, 1:500; Cell Signaling Technology, Danvers, MA, USA), rabbit monoclonal antibody against NMDAR2A (ab133265, 1:1000; Abcam, Cambridge, MA, USA). The membranes were incubated with goat anti-rabbit immunoglobulin G (31460, 1:8000; Thermo Fisher, West Grove, PA, USA). Then, the immunoreactivity was visualized using the SuperSignal West Femto Chemiluminescent Substrate kit (34094, Thermo Fisher Scientific). The relative intensities of the protein bands using ImageJ software (Zong et al., 2017). α-Tubulin and GAPDH were used as the internal standard.

### Immunohistochemistry

Detailed steps as described in our previous articles (Wu et al., 2010; Zhou et al., 2017). The cryosections were then incubated with rabbit polyclonal antibody against GABA_A_ alpha 1 (ab33299, 1:100; Abcam, Cambridge, MA, USA), rabbit polyclonal antibody against NMDAR2B (ab65783, 1:200; Abcam, Cambridge, MA, USA). The secondary antibodies (all from Invitrogen-Molecular Probes, Eugene, OR, USA; Zhou et al., 2017) were 555-conjugated donkey anti-rabbit IgG antibody (A31572, 1:1000) and 488-conjugated goat anti-rabbit IgG antibody (A11070, 1:500).

### Statistical Analysis

As previously described (Zhou et al., 2017), data are presented as the mean ± SEM. One-way analysis of variance test was used to compare the means among multiple groups. Student’s *t* test was used to compare the differences in means between two groups. The distributions of the amplitudes and frequency between the events were compared by Kolmogorov–Smirnov test. *p* < 0.05 was considered statistically significant.

## Results

### GABA Concentration-Response Relationships Were Determined

**Figure 1:**
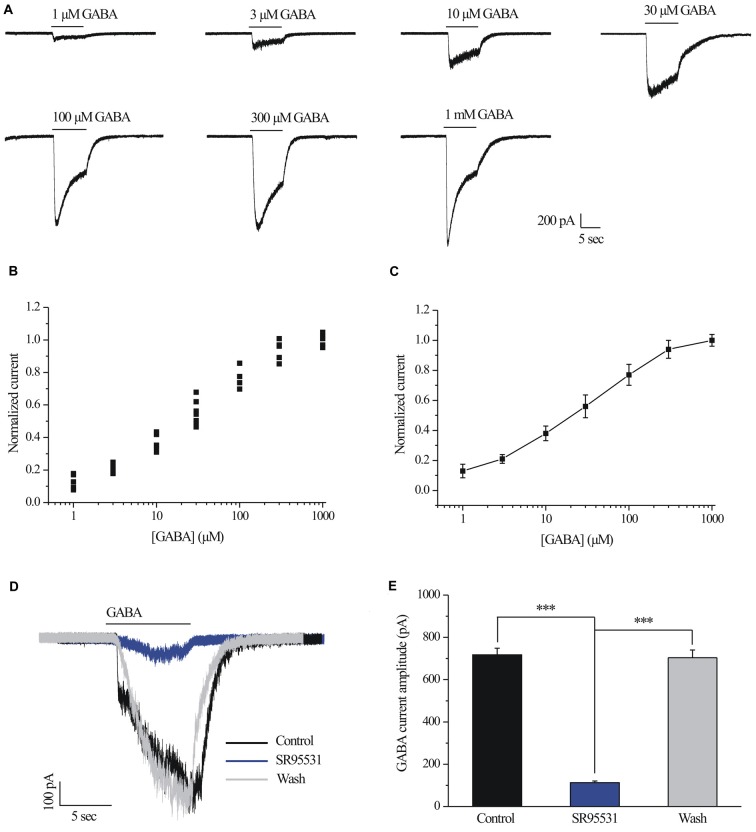
γ-aminobutyric acid (GABA) dose-response relationship of retinal ganglion cells (RGCs). **(A)** The concentrations of GABA are indicated above the current traces. The bars delineate the beginning and duration of GABA application (8 s). Vertical calibration bar, 200 pA; horizontal calibration bar, 5 s. **(B)** Scatter plot showing the GABA concentration-response relationship of RGCs. The data were normalized to the maximal current obtained with 1 mM GABA. **(C)** The mean ± SE of the cells in **(B)** were plotted vs. the GABA concentration. The line represents the best fit of the data to a sigmoidal function. **(D)** Representative GABA-induced whole-cell currents in control, in the presence of 10 μM SR95531, and after washing out SR95531. Vertical calibration bar, 100 pA; horizontal calibration bar, 5 s. Control and washout responses without SR95531 were measured at the beginning and end of each experiment, respectively. **(E)** Summarized data of the amplitude of GABA-evoked currents (*n* = 11). ***p < 0.001 (one-way analysis of variance). Holding potential was −70 mV. The results are expressed as the mean ± standard error.

Examples of current curves in response to seven GABA concentrations (ranging from 1 μM to 1 mM; Ramsey et al., 2007) are shown in Figure 1A. A pulse of GABA induced a dose-dependent inward current carried by chloride ions that became apparent at 30 μM GABA (*n* = 10; Figure 1A) and showed a maximal current of 1290 ± 49.49 pA with 1 mM GABA (*n* = 5; Figure 1A). Figure 1B illustrates the concentration-response amplitude relationship for the GABA_A_ receptor-mediated currents of RGCs from normal retinal slices fitted to the Hill equation with an EC50 of 28.7 ± 3.59 μM. Therefore, 30 μM was selected for subsequent experiments. For each RGC, inward currents induced by increasing concentrations of GABA were normalized to the maximal value obtained with 1 mM GABA (Figures 1B,C). GABA-evoked currents were completely and reversibly blocked by a specific GABA_A_ receptor antagonist SR95531 (10 μM; Figure 1D). The mean ± SE GABA-evoked currents amplitude was 717.5 ± 30.97 pA before SR95531 application and decreased to 113 ± 7.75 pA during SR95531 (*n* = 11, *p* < 0.001; Figure 1E). The GABA-elicited currents reflect almost exclusive activation of the GABA_A_ receptors.

### Chronic Glaucoma Altered the GABA-Induced Current Amplitude, and This Change Was Prevented by PNU-282987

**Figure 2:**
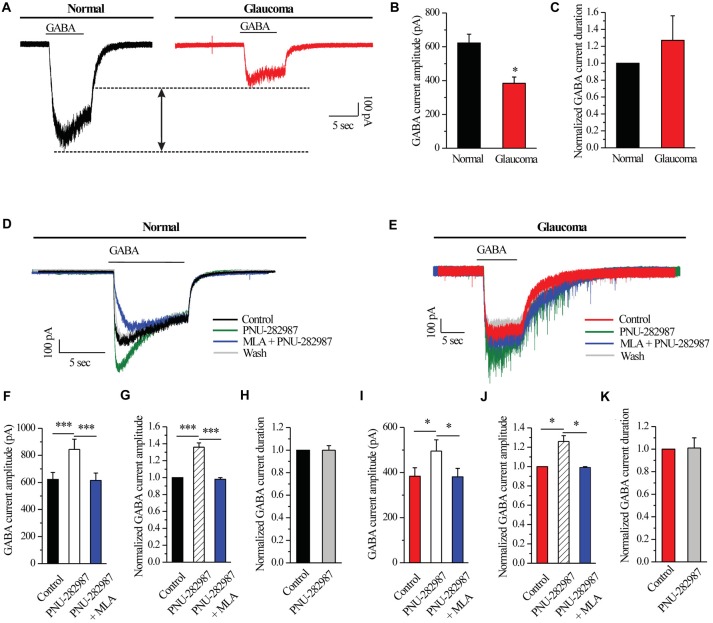
PNU-282987 modulated GABA-elicited whole-cell currents in RGCs of the rat retina slice. **(A)** The membrane current responses elicited by GABA (30 μM) from a control and glaucomatous retinal ganglion cell (RGC). **(B)** Bar graph illustrating the significant differences in the peak amplitudes of the responses recorded from normal (*n* = 11) and glaucomatous (*n* = 4) RGCs. **(C)** Summarized data of the duration of GABA-evoked currents (*n* = 5). **(D)** In normal RGCs, representative GABA-induced currents in control, in the presence of 10 μM PNU-282987, during 100 nM methyllycaconitine (MLA) + 10 μM PNU-282987 application and after washing out the study drugs. Vertical calibration bar, 100 pA; horizontal calibration bar, 5 s. **(E)** In glaucomatous RGCs, representative GABA-induced currents in control, in the presence of 10 μM PNU-282987, during 100 nM MLA + 10 μM PNU-282987 application and after washing out study drugs. Vertical calibration bar, 100 pA; horizontal calibration bar, 5 s. **(F)** Statistical analysis of the GABA current amplitude from ten normal RGCs. **(G)** Normalized GABA current amplitude from ten normal RGCs. **(H)** Normalized GABA current duration from ten normal RGCs. **(I)** Statistical analysis of GABA current amplitude from ten glaucomatous RGCs. **(J)** Normalized GABA current amplitude from ten glaucomatous RGCs. **(K)** Normalized GABA current duration from ten glaucomatous RGCs. *p < 0.05, ***p < 0.001 (Student’s paired *t* test). The results are expressed as the mean ± standard error.

A fast inward current could be induced by local pressure application of GABA (30 μM) for 8 s and could be reproducibly elicited at 3-min intervals. As shown in examples of the GABA-elicited membrane current recorded from an RGC from a normal and glaucomatous retina in Figure 2A, the GABA-elicited current from glaucomatous RGCs was significantly smaller (383.75 ± 37.6 pA, *n* = 4) than that from control cells (622.5 ± 51.7 pA, *n* = 11; *p* < 0.05; Figure 2B). However, the mean GABA current duration was not significantly different between the control and glaucomatous RGCs, with the duration in the glaucomatous RGCs equivalent to 127% ± 29% of that in the control (*n* = 5, *p* = 0.209; Figure 2C).

Next, RGCs were examined with patch-clamp techniques in order to identify the effects of α7-nAChR on GABA_A_ receptors. In normal RGCs, activation of α7-nAChR by PNU-282987, a selective α7-nAChR agonist, led to an enhancement of GABA receptor function, as shown in Figure 2D. Pre-applied PNU-282987 caused a significant increase in the amplitude of the GABA-induced current (622.5 ± 51.7 pA vs. 844 ± 75.20 pA, *n* = 10, *p* < 0.001; Figure 2F), equivalent to 136% ± 5% of the control level (*n* = 10, *p* < 0.001; Figure 2G). A highly selective α7-nAChR antagonist, MLA blocked the effects of PNU-282987 on the amplitude of the GABA-induced current (Figures 2D,F,G). However, the mean GABA current duration was not significantly different between the control and the PNU-282987-treated groups (100% ± 4% of the control level, *n* = 10, *p* = 0.989; Figure 2H).

Similar changes were observed in the glaucomatous retina (Figure 2E). Administration of PNU-282987 (10 μM) significantly increased the amplitude of GABA-induced currents in the glaucomatous retina (383.75 ± 37.60 pA vs. 495 ± 50.04 pA, *n* = 4, *p* < 0.05; Figure 2I) with an increase to 126% ± 6% of the normal level (*n* = 5, *p* < 0.05; Figure 2J). The changes of PNU-282987 on the amplitude of the GABA-induced current were blocked by MLA (100 nM; Figures 2E,I,J). However, the mean GABA current duration was not significantly different between the control and the PNU-282987-treated groups (100% ± 9% of the control level, *n* = 5, *p* = 0.921; Figure 2K). Change in the GABA response was fully reversed after washout of PNU-282987. These results from patch-clamp recordings of RGCs revealed profound changes in the GABA_A_ receptor properties under glaucoma conditions.

### Chronic Glaucoma Altered GABA_A_ Receptor Protein Expression, and These Changes Were Prevented by PNU-282987

**Figure 3:**
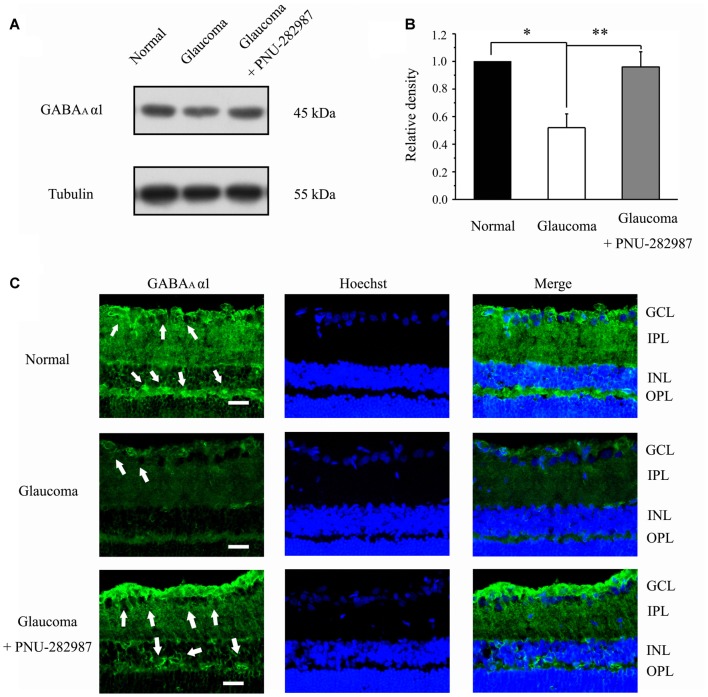
Effect of PNU-282987 on the expression levels of the GABA receptor subunit GABAAα1 in the retina. **(A)** Immunoblot analysis of GABAAα1 in the retinas of control and glaucomatous rats before and after PNU-282987 injection. Full-length blots are presented in Supplementary Figure S1. **(B)** Quantitative analysis of GABAAα1 expression. **(C)** Immunohistochemical analysis of the rat retina with antibodies against GABAAα1 labeled in ganglion cell layer (GCL; arrow) and outer plexiform layer (OPL; arrow) in the normal, glaucoma and glaucoma + PNU-282987 conditions. GCL, ganglion cell layer; IPL, inner plexiform layer; INL, inner nuclear layer; OPL, outer plexiform layer. Scale bar: 15 μm. The data are presented as the mean ± standard error of four samples for each experiment. *p < 0.05, **p < 0.01 (one-way analysis of variance).

To investigate whether electrophysiological results were associated with changes of GABA_A_α1 subunit of GABA receptors, we next examined GABA_A_α1 protein expression by immunoblot analysis in control and glaucomatous rats before and after PNU-282987 treatment. PNU-282987 was injected intravitreally on day 0 (the day of EVC) and every 7 days thereafter (Zhou et al., 2017). The level of GABA_A_α1 protein expression in glaucomatous rats was significantly lower than in control rats (52% ± 10%, *n* = 4, *p* < 0.05; Figures 3A,B), but the level was increased after PNU-282987 treatment (96% ± 11%, *n* = 4, *p* < 0.05; Figures 3A,B). Immunohistochemical analysis revealed that immunoreactivity of GABA_A_α1 protein was low in glaucomatous rats but was significantly increased, especially in the outer plexiform layer (OPL) and the ganglion cell layer (GCL; Li et al., 2017), in PNU-282987-treated glaucomatous rats at 3 weeks (Figure 3C). These results suggest that PNU-282987 exerts a direct neuroprotective effect, at least partly, by enhancing the GABA_A_ receptor expression and thus suppressing excitotoxicity.

### Chronic Glaucoma or PNU-282987 Did Not Change the Glutamatergic mEPSCs of RGCs

**Figure 4:**
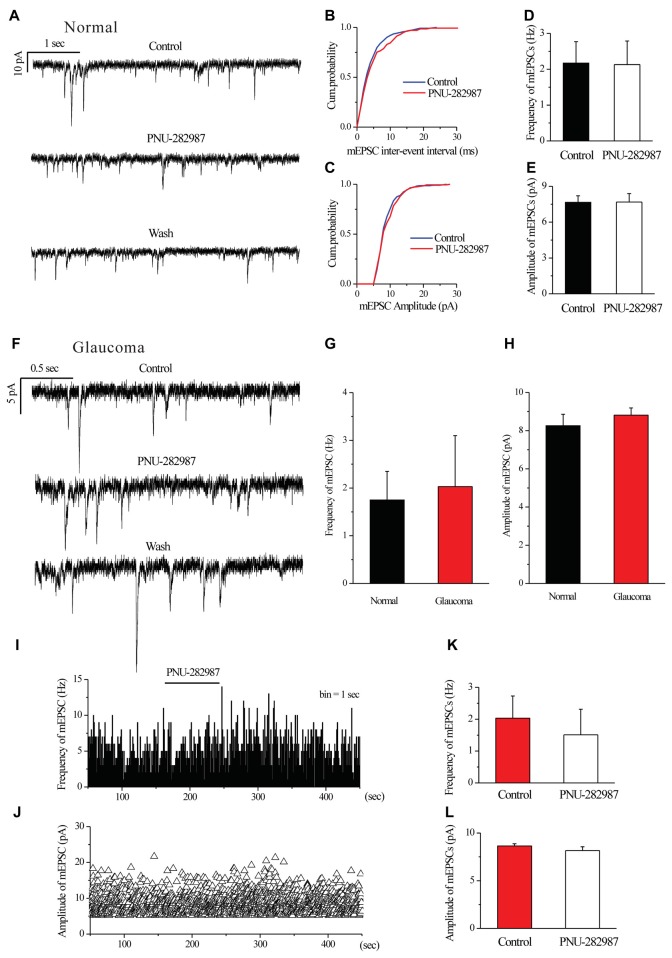
Ocular hypertension or PNU-282987 did not cause a significant frequency or amplitude change in the miniature glutamatergic excitatory postsynaptic currents (mEPSCs) of RGCs. **(A)** Recording from a representative normal rat experiment during control conditions, during PNU-282987 application (10 μM) and during recovery. Vertical calibration bar, 10 pA; horizontal calibration bar, 1 s. **(B,C)** Cumulative probability plots of mEPSC inter-event interval **(B)** and amplitude **(C)** before and during application of PNU-282987 (*n* = 6, Kolmogorov–Smirnov test).** (D,E)** Summarized data of the frequency **(D)** and amplitude **(E)** of the mEPSCs in normal RGCs (*n* = 6). **(F)** Recording from a representative glaucomatous rat experiment during control conditions, during PNU-282987 application (10 μM) and during recovery. Vertical calibration bar, 5 pA; horizontal calibration bar, 0.5 s. **(G,H)** Bar graph illustrating the frequency **(G)** and peak amplitude **(H)** of the responses recorded from normal (*n* = 4 in frequency; *n* = 6 in amplitude) and glaucomatous (*n* = 5) RGCs. **(I,J)** Frequency (1-s bin) and amplitude histograms of the mEPSCs of the trace in **(F)**.** (K,L)** Summarized data of the frequency **(K)** and amplitude **(L)** of the mEPSC in glaucomatous RGCs (*n* = 6). The results in **(D,E,G,H,K,L)** are expressed as the mean ± standard error.

We further examined if glutamatergic synaptic inputs in glaucomatous retinal slices were affected by examining the miniature glutamatergic excitatory postsynaptic currents (mEPSCs) of RGCs. Figures 4A,F illustrate sweeps of mEPSCs from an RGC from a control and a glaucomatous rat, showing that the mean mEPSC frequency and amplitude were not significantly different (Figure 4G, 1.75 ± 0.60 Hz in control, *n* = 4, vs. 2.03 ± 1.07 Hz in glaucoma, *n* = 5, *p* = 0.838; Figure 4H, 8.26 ± 0.60 pA in control, *n* = 6, vs. 8.81 ± 0.38 pA in glaucoma, *n* = 5, *p* = 0.481).

In normal RGCs, PNU-282987 did not change the frequency and the amplitude of mEPSCs in the presence of 1 μM TTX (Figures 4A–E). The effects of PNU-282987 on the cumulative distributions of the frequency and amplitude of mEPSCs (Kolmogorov–Smirnov test) are shown in Figure 4B (*n* = 6, *p* > 0.05) and 4C (*n* = 6, *p* > 0.05), respectively. The mean mEPSC frequency and amplitude were not significantly different between the control and glaucomatous RGCs (Figure 4D, 2.17 ± 0.60 Hz vs. 2.13 ± 0.66 Hz, *n* = 3, *p* = 0.898; Figure 4E, 7.66 ± 0.55 pA vs. 7.68 ± 0.72 pA, *n* = 4, *p* = 0.948).

Similarly, PNU-282987 (10 μM) did not change the frequency or amplitude of glutamatergic mEPSCs in glaucomatous retinas (Figures 4F,I–L). The time course of the change in the frequency and amplitude of glutamatergic mEPSCs in response to PNU-282987 application in a representative RGC are shown in a frequency histogram (Figure 4I) and plot of the running amplitude (Figure 4J). The mean mEPSC frequency and amplitude were not significantly different between the control and glaucomatous RGCs (Figure 4K, 2.03 ± 0.70 Hz vs. 1.51 ± 0.80 Hz, *n* = 6, *p* > 0.05; Figure 4L, −8.64 ± 0.23 pA vs. 8.17 ± 0.39 pA, *n* = 6, *p* > 0.05).

### Chronic Glaucoma Altered the NMDA Current Amplitude, and This Change Was Prevented by PNU-282987

**Figure 5:**
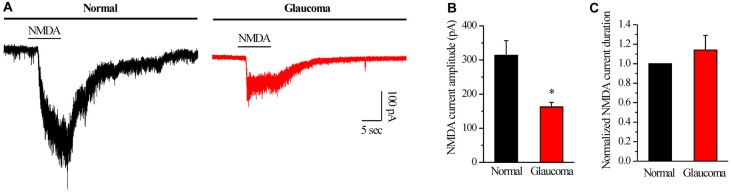
N-methyl-D-aspartate (NMDA)-elicited whole-cell currents in RGCs of the normal and glaucomatous rat retina slice.** (A)** The membrane current responses elicited by NMDA (100 μM) from normal and glaucomatous RGCs. **(B)** Bar graph illustrating the significant differences in the peak amplitudes of the responses recorded from normal (*n* = 5) and glaucomatous (*n* = 4) RGCs.** (C)** Summarized data of the duration of NMDA-evoked currents (*n* = 4). *p < 0.05 (Student’s paired *t* test). Holding potential was −70 mV. Vertical calibration bar, 100 pA; horizontal calibration bar, 5 s. The results are expressed as the mean ± standard error.

We further examined if NMDAR function was changed in glaucomatous retinal slices by examining the NMDA-elicited whole-cell currents of RGCs in the retina. The I_NMDA_ was evoked once every 3 min using an 8-s exposure to NMDA (with glycine). As shown in representative current traces in Figure 5A, the I_NMDA_ recorded from glaucomatous RGCs was significantly smaller (162.5 ± 13.15 pA, *n* = 4) than that from control cells (314 ± 42.38 pA, *n* = 5; *p* < 0.05; Figure 5B). However, the mean I_NMDA_ duration was not significantly different between the control and glaucomatous RGCs, with the duration in the glaucomatous RGCs equivalent to 131% ± 27% of the duration in the control (*n* = 4, *p* = 0.306; Figure 5C).

**Figure 6:**
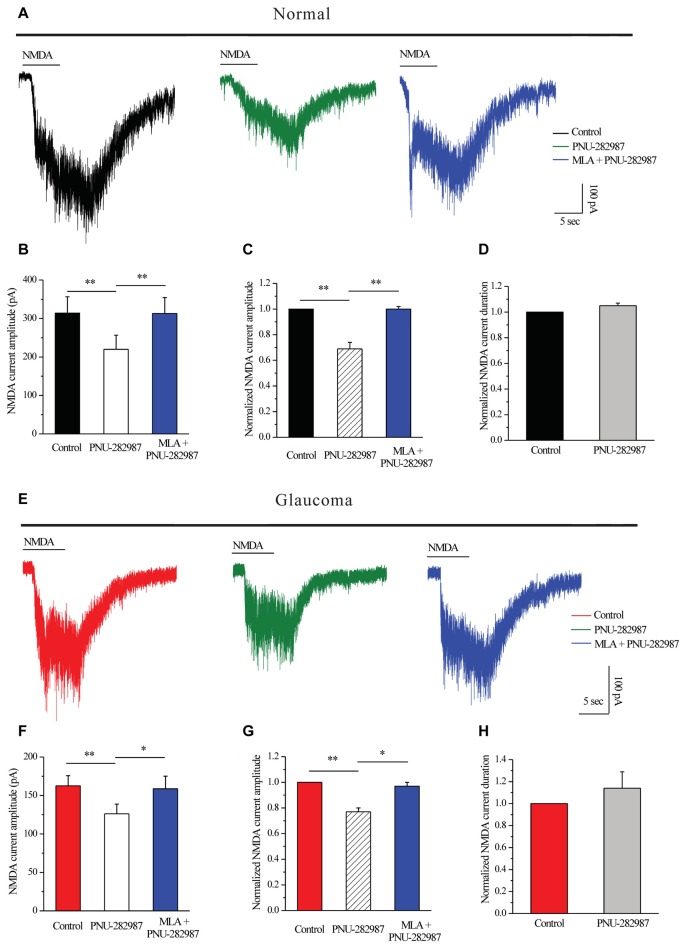
PNU-282987 modulated NMDA-elicited whole-cell currents in RGCs of the rat retina slice. **(A)** In normal RGCs, representative NMDA-induced currents in control, in the presence of 10 μM PNU-282987 and 100 nM MLA + 10 μM PNU-282987. **(B)** Summarized data of NMDA current amplitude from five normal RGCs. **(C)** Normalized NMDA current amplitude from five normal RGCs.** (D)** Normalized NMDA current duration from five normal RGCs. **(E)** In glaucomatous RGCs, representative NMDA-induced currents in control, in the presence of 10 μM PNU-282987 and during 100 nM MLA + 10 μM PNU-282987 application. **(F)** Statistical analysis of NMDA current amplitude from four glaucomatous RGCs. **(G)** Normalized NMDA current amplitude from four glaucomatous RGCs.** (H)** Normalized NMDA current duration from four glaucomatous RGCs. Vertical calibration bar, 100 pA; horizontal calibration bar, 5 s. *p < 0.05, **p < 0.01 (one-way analysis of variance). The results are expressed as the mean ± standard error.

Next, we tested whether PNU-282987 could modulate NMDARs. PNU-282987 was continuously bath-applied for 5 min after a 5-min stable baseline recording. The amplitude of the I_NMDA_ in RGCs was reversibly decreased after PNU-282987 pretreatment (314 ± 42.38 pA vs. 220 ± 36.84 pA, *n* = 5, *p* < 0.01; Figures 6A,B), equivalent to 69% ± 5% of the control level (*n* = 5, *p* < 0.01; Figures 6A,C). However, the mean I_NMDA_ duration was not significantly different between the control and the PNU-282987-treated groups (105% ± 2% of the control level, *n* = 5, *p* = 0.066; Figure 6D). After preincubation with MLA (100 nM), PNU-282987 failed to alter the I_NMDA_ (Figures 6A–C).

Similar changes were observed in the glaucomatous retina (Figure 6E) where administration of PNU-282987 (10 μM) caused a decrease in peak I_NMDA_ (162.5 ± 13.15 pA vs. 126.25 ± 12.48 pA, *n* = 4, *p* < 0.01; Figure 6F) to 77% ± 3% of the control level (*n* = 4, *p* < 0.01; Figure 6G). However, the I_NMDA_ duration was not significantly different between the control and the PNU-282987-treated groups (114% ± 15% of the control level, *n* = 4, *p* = 0.403; Figure 6H). NMDAR function was fully recovered after washout of PNU-282987. In the presence of MLA (100 nM), PNU-282987 failed to alter the I_NMDA_ (Figures 6E–G). These results from patch-clamp recordings of RGCs revealed profound changes in the NMDAR properties under glaucoma conditions.

**Figure 7:**
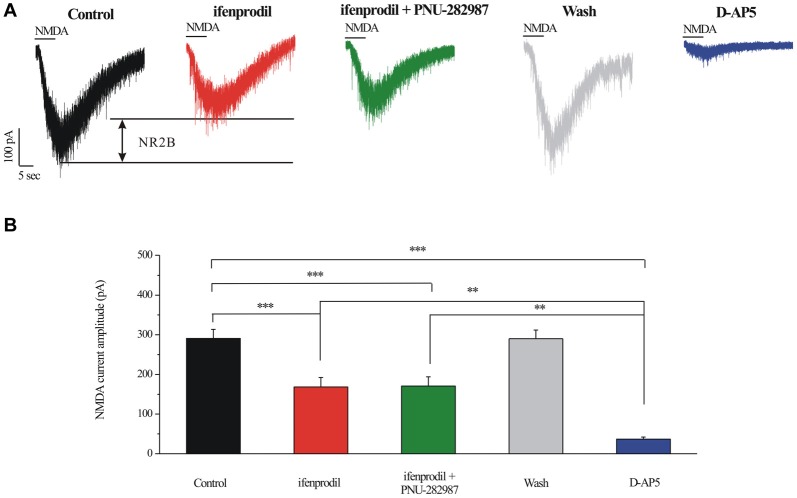
Effect of ifenprodil, PNU-282987 and D-AP5 on NMDA-elicited currents in RGCs of the rat retina slice. **(A)** Representative NMDA-induced currents showing the amplitude of the NMDA-evoked currents (recorded in 0 Mg^2+^ ACSF) in control, in the presence of 10 μM ifenprodil, ifenprodil + PNU-282987, after washing out drugs and during D-AP5 (50 μM). Vertical calibration bar, 100 pA; horizontal calibration bar, 5 s. **(B)** Summarized data of the amplitude of NMDA-evoked currents (*n* = 6). **p < 0.01, ***p < 0.001 (one-way analysis of variance). Holding potential was −70 mV. The results are expressed as the mean ± standard error.

I_NMDA_ were partially blocked by a selective NR2B antagonist ifenprodil (10 μM; Figures 7A,B). The mean ± SE I_NMDA_ amplitude was 290.83 ± 22.78 pA before ifenprodil application and decreased to 168.33 ± 23.83 pA during ifenprodil (*n* = 6, *p* < 0.001; Figure 7B). In the presence of ifenprodil, bath-applied PNU-282987 did not change the peak amplitude of I_NMDA_ (168.33 ± 23.83 pA vs. 170.83 ± 23.18 pA, *n* = 6, *p* = 0.490; Figure 7B), the change in the NMDA response was fully reversed after washout of ifenprodil + PNU-282987. The robust inward current was blocked by D-AP5, a specific NMDAR antagonist, the mean I_NMDA_ in ifenprodil and D-AP5 group was significantly different (168.33 ± 23.83 pA vs. 36.67 ± 5.43 pA, *n* = 6, *p* < 0.01; Figures 7A,B). The I_NMDA_ in ifenprodil + PNU-282987-treated slices was similar to that in ifenprodil-treated slices, indicating that PNU-282987 does not decrease NR1/NR2A-elicited currents (Figures 7A,B).

### Chronic Glaucoma Altered NMDA Protein Expression, and These Changes Were Prevented by PNU-282987

**Figure 8:**
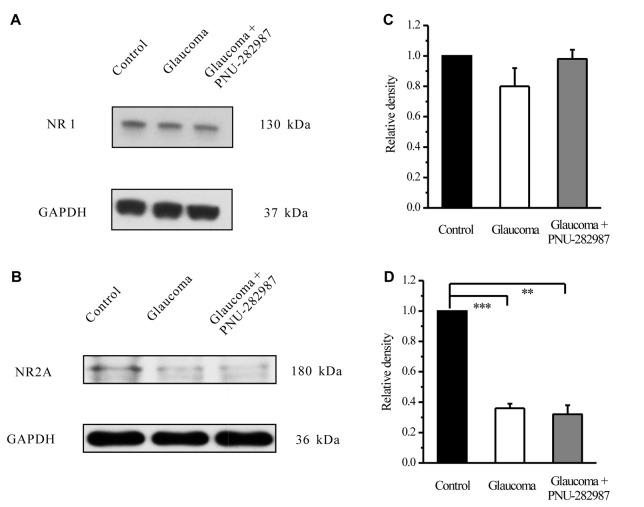
Effect of PNU-282987 on NR1/NR2A expression levels in the retina. **(A)** Immunoblot analysis of NR1 in the retinas of control and glaucomatous rats before and after PNU-282987 injection. **(B)** Quantitative analysis of NR1 expression. **(C)** Immunoblot analysis of NR2A in the normal, glaucoma and glaucoma + PNU-282987 conditions. **(D)** Quantitative analysis of NR2A expression. The data are presented as the mean ± standard error of four samples for each experiment. **p < 0.01, ***p < 0.001 (one-way analysis of variance). Full-length blots are presented in Supplementary Figure S1.

To explore the possible interactions of α7-nAChR and NMDARs in glaucoma, we examined the effects of PNU-282987 on the expression levels of the NMDAR subunit NR1, NR2A and NR2B in retinas. In the Western blot analysis, the immunoreactive bands of the NR1 protein, detected at approximately 130 kDa (Figure 8A). However, the mean NR1 protein level in control and glaucomatous retinas was not significantly different, equivalent to 80% ± 13% of the control level (*n =* 4, *p =* 0.179; Figure 8B). And that PNU-282987 failed to alter the NR1 protein level. The immunoreactive bands of the NR2A protein, detected at approximately 180 kDa (Figure 8C), indicated significantly lower NR2A expression levels in glaucomatous retinas (36% ± 3%, *n* = 4, *p* < 0.001; Figure 8D) than in control. However, PNU-282987 did not change NR2A protein expression in glaucomatous retinas.

**Figure 9:**
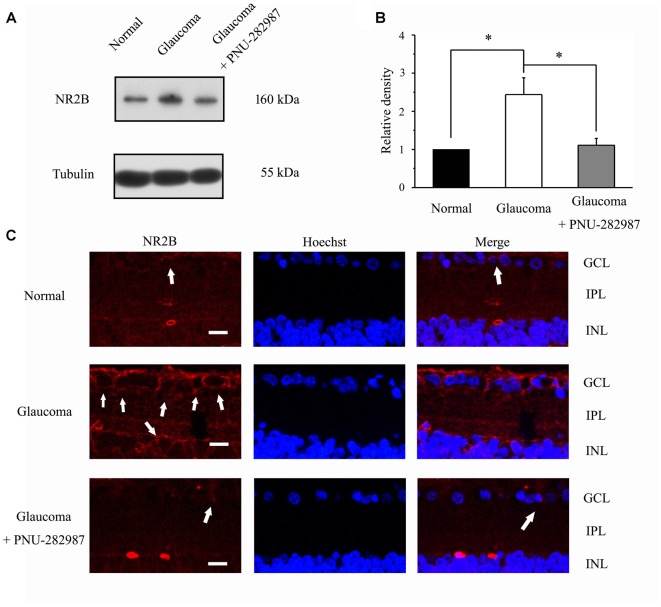
Effect of PNU-282987 on NR2B expression levels in the retina.** (A)** Immunoblot analysis of NR2B in the retinas of control and glaucomatous rats before and after PNU-282987 injection. Full-length blots are presented in Supplementary Figure S1.** (B)** Quantitative analysis of NR2B expression. **(C)** Confocal images showing the distribution of NR2B labeled in the GCL (arrow) and INL (arrow) in the normal, glaucoma and glaucoma + PNU-282987 conditions. GCL, ganglion cell layer; IPL, inner plexiform layer; INL, inner nuclear layer. Scale bar: 30 μm. The data are presented as the mean ± standard error of five samples for each experiment. *p < 0.05 (one-way analysis of variance).

The immunoreactive bands of the NR2B protein, detected at approximately 160 kDa (Figure 9A), indicated significantly higher NR2B expression levels in glaucomatous retinas (144% ± 44%, *n* = 5, *p* < 0.05; Figure 9B) than in control. Furthermore, PNU-282987 decreased NR2B protein expression in glaucomatous retinas to 48% ± 12% of that in vehicle-treated retinas (*n* = 5, *p* < 0.05). Immunofluorescence revealed that NR2B expression was found in the inner nuclear layer (INL) and GCL (Figure 9C) and was higher in glaucomatous rats than in control. Furthermore, administration of PNU-282987 decreased NR2B expression (Figure 9C). These results suggest that PNU-282987 reduces NR2B overexpression due to glutamate neurotoxicity in the retina. NMDAR-mediated currents can be directly inhibited by PNU-282987, possibly through a direct interaction with the NR2B subunit of the NMDAR.

## Discussion

The present study demonstrates that activation of α7-nAChR by PNU-282987 reversibly enhances GABA_A_ receptor and depresses NMDAR expression and function. Taken together, our data provide insights into how an α7-nAChR agonist modulate postsynaptic amino acid receptors, which may help to uncover the mechanisms of α7-nAChR protection in glaucoma.

The modulation of NMDAR and GABA_A_ by α7-nAChR agonists may be a new therapeutic schedule of cholinergic innervation in glaucoma (Shen et al., 2016). α7-nAChR in the retinas is important to regulate synaptic connection and neuronal excitability because cholinergic receptors are widely expressed on terminals of ON cone bipolar cells and amacrine cells containing GABA (Liu et al., 2006; Dmitrieva et al., 2007).

The α7 nAChRs were frequently expressed on the inner retina and affect ganglion cell responses directly and indirectly in animal models (Dougherty et al., 2003; Dmitrieva et al., 2007). Activation of α7-nAChR on RGCs can directly affect the responses of these cells (Dougherty et al., 2003). Activation of α7-nAChR on GABAergic and glycinergic amacrine cells causes the depolarization of amacrine cells, increasing the release of inhibitory neurotransmitters and inhibiting indirectly the ganglion cell responses (Dmitrieva et al., 2007).

The effects of α7-nAChR on GABAergic signaling reported here are different from previous reports that α7-nAChR agonists depress GABA- or muscimol-evoked currents in hippocampal interneurons (Zhang and Berg, 2007). In dissociated chick ciliary ganglion (CG) neurons, activation of α7-nAChR diminishes GABA_A_ receptor responses (Zhang and Berg, 2007). In contrast to previous reports, we demonstrate that the α7-nAChR agonist enhanced GABA_A_ receptor-mediated whole-cell currents in RGCs. Together, these data suggest that α7-nAChR can have different effects depending on the different preparations used in these experiments (e.g., dopaminergic neurons from the substantia nigra pars compacta vs. neurons from CG and hippocampal interneurons) from different species (e.g., Wistar rat retinal slices vs. dissociated chick embryonic neurons; Zong et al., 2017).

In the CNS, GABA_A_ receptors are responsible for mediating the inhibitory effects of the endogenous GABAergic inhibitory system (Ramsey et al., 2007). Activation of GABA_A_ receptors increases chloride ion conductance, inducing hyperpolarization and reducing cell excitability (Shen et al., 2016). Therefore, GABA_A_ receptor system can regulate intrinsic characteristics of excitability in cells. Our observations provided the first evidence that PNU-282987 is sufficient to enhance postsynaptic GABA_A_ currents onto RGCs in the retina, which may in turn reduce the excitability of RGCs due to the increase in hyperpolarization of the GABAergic responses. In other words, PNU-282987 may produce its neuroprotective effects via hyperpolarizing RGCs in glaucoma.

Combined with our previous study, our data indicate a reduction in GABAergic activity (presynaptic GABA release and postsynaptic GABA_A_ receptor expression and function) in glaucomatous rats, potentially due to a reduction in numbers of GABAergic amacrine cells and their synaptic terminal boutons on RGCs and a reduction in the GABA_A_ receptor activity in RGCs. The decrease in the amplitude of GABA_A_ receptor-mediated current and the reduction in the immunoreactivity of the GABA receptor subunit GABA_A_α1 point to a significant loss of GABAergic system function in glaucomatous RGCs.

The NMDAR can be highly permeable to Ca^2+^ ions, and its overactivation in disease conditions can lead to intracellular Ca^2+^ overload, leading to cell death (Sattler and Tymianski, 2001; Dong et al., 2008). NMDARs include NR1 and NR2 subunits. The NR2A–D subunits are the important functional unit of NMDARs (Bai et al., 2013b). Synaptic NR1/NR2A-containing NMDARs activity tend to promote cell survival, while the extrasynaptically localized NR2B subunit activation is usually associated with cell death (Hardingham et al., 2002; Kim et al., 2005). Activation of NR2A, or inhibition of NR2B contributed to the relief of sevoflurane neurotoxicity. Moreover, sevoflurane-induced neuronal apoptosis could be prevented by application of NR2B inhibitor ifenprodil (Kaufman et al., 2012; Wang et al., 2016). In DBA/2J mice, reports have shown that high IOP can increase the expression of NR2B subunits. In contrast, there are no difference in the protein levels of NR1 and NR2A subunits of the retinas of DBA/2J compared to C57BL/6 mice (Dong et al., 2013). In contrast to previous reports, we demonstrate that an elevated IOP-induced increase in expression of the NR2B subunits and decrease in expression of the NR2A subunits of NMDARs, and the protein levels of the NR1 subunits are not affected. Administration of PNU-282987 decreased NR2B overexpression but not changed NR1/NR2A protein levels.

Pharmacological inhibition of the NR2B subunit of NMDARs attenuated RGC loss in glutamate aspartate transporter-deficient (GLAST) mice, a model of normal-tension glaucoma (NTG; Bai et al., 2013a). NR2B is included in the loss of RGCs evoked by excitotoxicity. The interaction between NR2B and α7-nAChR may have the neuroprotective effect. Decreased glutamate toxicity through downregulation of NMDAR expression and function following α7-nAChR stimulation could be another mechanism underlying the neuroprotective effects of PNU-282987, in parallel with the up-regulation of GABA_A_ receptor expression and function.

Interestingly, the smaller I_NMDA_ from the “glaucoma” RGCs than that elicited from the “normal” cells might represent a compensatory mechanism to reduce the excitatory synaptic strength following the loss of function in inhibitory regulation (Ortinski et al., 2006). Altered receptor subunit composition in the postsynaptic membrane of glaucomatous rat RGCs may be one of the possible reasons. Previous studies have shown that NR2A subunits expression may be an important factor in regulating NMDAR EPSC duration and synaptic plasticity. In the postnatal neocortex neurons, cells expressing NR2A subunit mRNA had faster NMDAR EPSCs than cells not expressing this subunit (Zong et al., 2017). NR2A-containing NMDARs inhibitors decresed ~30% of the whole-cell current in rat pyramidal neurons (Volkmann et al., 2016). In our study, the reduction of NR2A expression may be responsible for the smaller I_NMDA_ under glaucoma conditions.

## Conclusion

The activation of α7-nAChR modulates neuronal circuit activity and cellular excitability. This effect depends on positive modulation of the GABA_A_ receptor, as well as negative modulation of NMDAR.

## Author Contributions

JW and SZ designed the experiments, modified the manuscript; XZhou and YZ did the experiments and wrote the manuscript; RZ and XZhang analyzed the data; XS modified the manuscript.

## Conflict of Interest Statement

The authors declare that the research was conducted in the absence of any commercial or financial relationships that could be construed as a potential conflict of interest.
