# Supplementary material for: Differential Modulation of GABAA and NMDA Receptors by an α7-nicotinic Acetylcholine Receptor Agonist in Chronic Glaucoma
Source: Front Mol Neurosci. 2017 Dec 18;10:422. doi: 10.3389/fnmol.2017.00422 (PMC5741651; doi:10.3389/fnmol.2017.00422)
Supplement: Supplementary file 1 [file Data_Sheet_1.docx]

Supplementary Material

**Differential modulation of GABA_A_ and NMDA receptors by α7-nicotinic acetylcholine receptor agonist in chronic glaucoma**

Xujiao Zhou^1,2,3a^, Yuan Zong^2,3,4a^, Rong Zhang^1^, Xuejin Zhang^1^, Shenghai Zhang^1^, Jihong Wu^1,2,3,4^*, Xinghuai Sun^1,2,3,4^

*** Correspondence:** Jihong Wu: [jihongwu@fudan.edu.cn](mailto:jihongwu@fudan.edu.cn)

Supplementary Figure


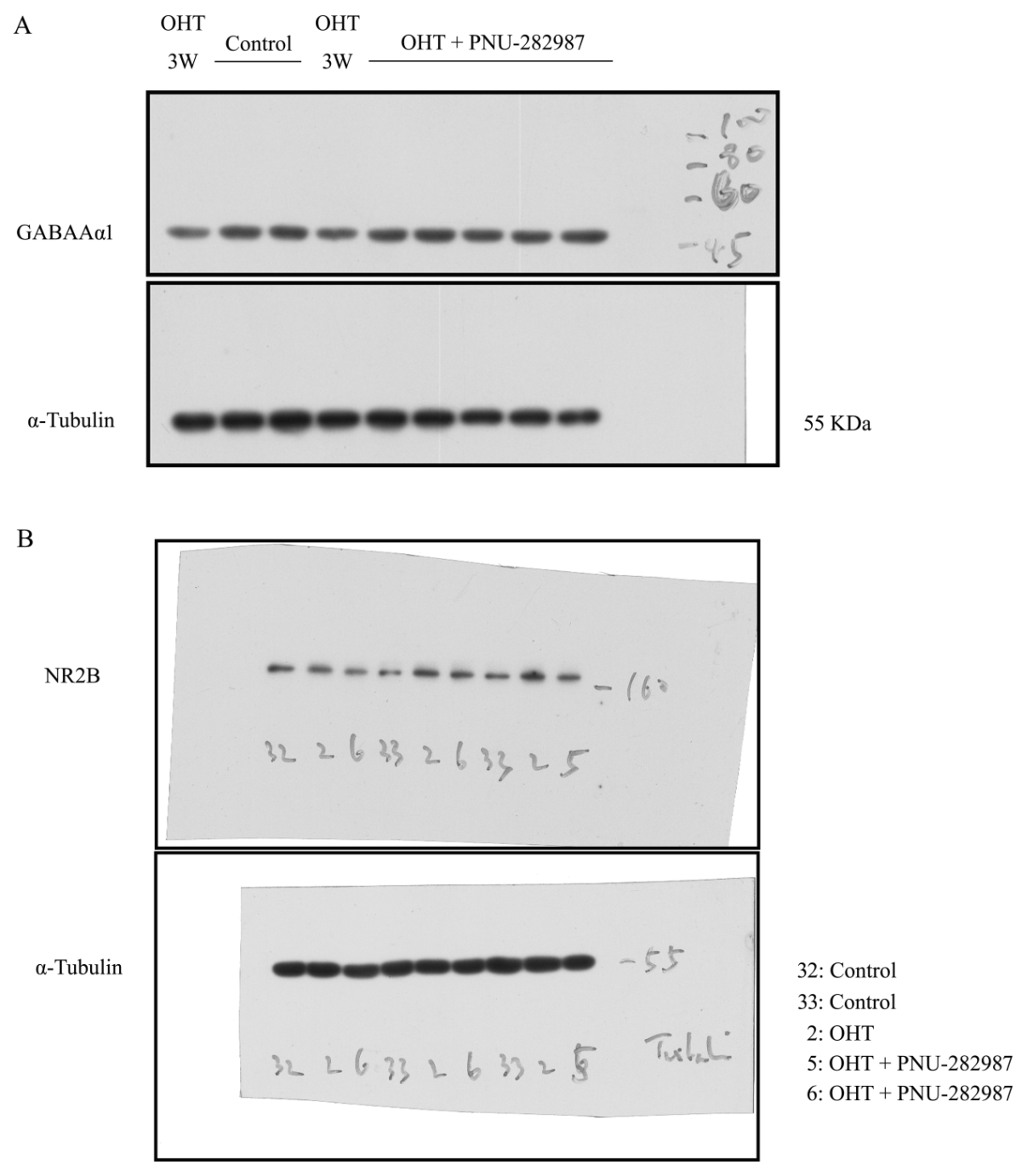


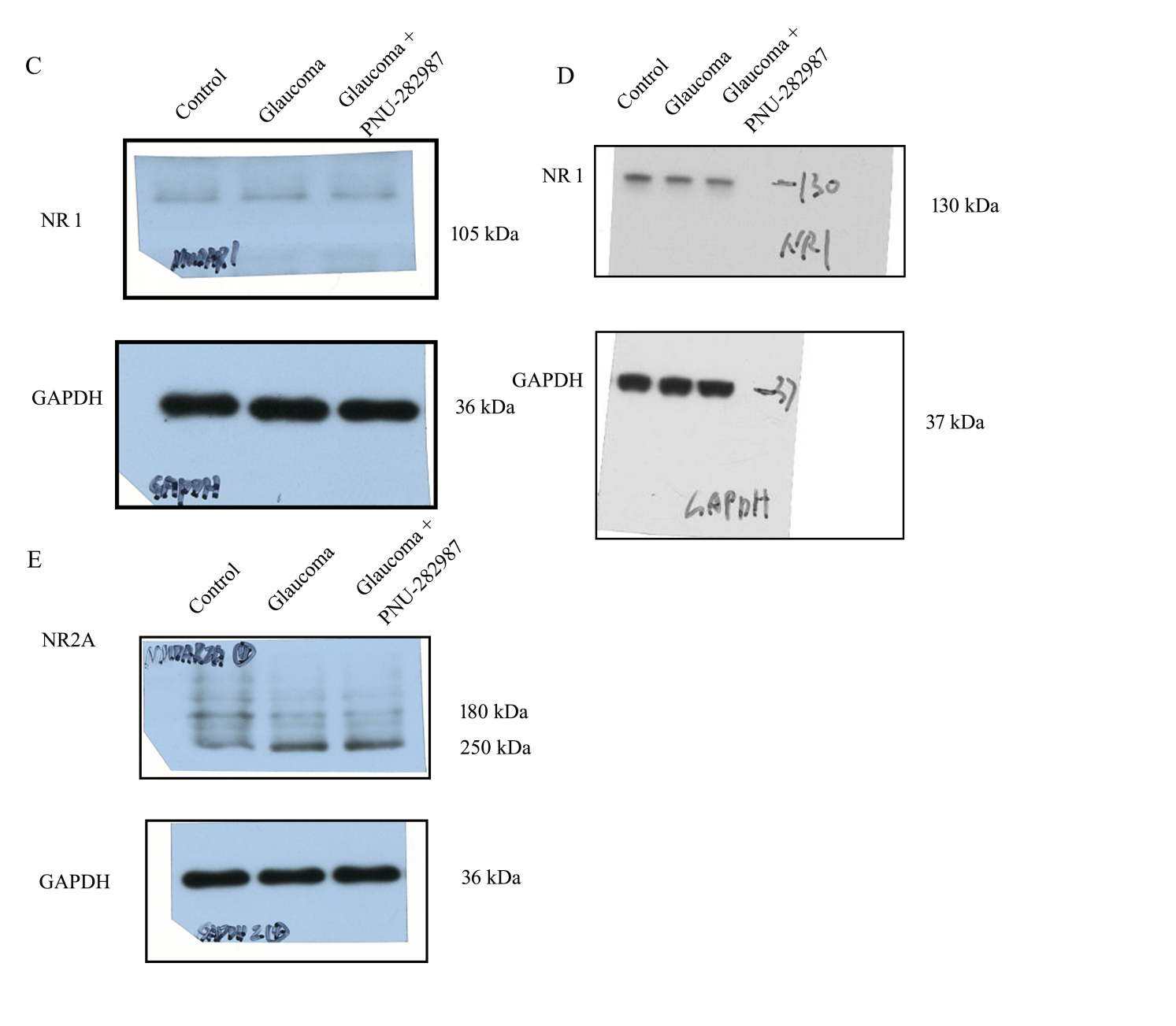


**Supplementary Figure S1.** Full-length blots of Figure 3A, Figure 9A, Figure 8A and Figure 8C. **(A)** Western blotting analysis of protein expression in control and glaucomatous retinas before and after the injection of PNU-282987 at 3 weeks after episcleral vein cauterization (EVC). Full-length blots of Figure 3A, showing the expression of GABA_A_α1 and α-tubulin. **(B)** Western blot showing the protein level of NR2B subunit in control and glaucomatous retinas before and after PNU-282987 treatment at 3 weeks after EVC. Full-length blots of Figure 9A, showing the expression of NR2B and α-tubulin. **(C)** Western blotting analysis of NR1 (ab68144) protein expression in the normal, glaucoma and glaucoma + PNU-282987 conditions at 3 weeks after EVC. **(D)** Western blotting analysis of NR1 (#5704) protein expression in the normal, glaucoma and glaucoma + PNU-282987 conditions at 3 weeks after EVC. Full-length blots of Figure 8A, showing the expression of NR1 and GAPDH. **(E)** Western blotting analysis of NR2A protein expression in control and glaucomatous retinas before and after the injection of PNU-282987 at 3 weeks after EVC. Full-length blots of Figure 8C, showing the expression of NR2A and GAPDH. W, week; OHT, ocular hypertension.
